# Foetal Loss and Enhanced Fertility Observed in Mice Treated with Zidovudine or Nevirapine

**DOI:** 10.1371/journal.pone.0107899

**Published:** 2014-09-18

**Authors:** Chika K. Onwuamah, Oliver C. Ezechi, Ebiere C. Herbertson, Rosemary A. Audu, Innocent A. O. Ujah, Peter G. C. Odeigah

**Affiliations:** 1 Human Virology Laboratory, Nigerian Institute of Medical Research, Lagos, Nigeria; 2 Clinical Sciences Division, Nigerian Institute of Medical Research, Lagos, Nigeria; 3 Department of Cell Biology & Genetics, Faculty of Science, University of Lagos, Akoka, Lagos, Nigeria; University of Missouri-Kansas City, United States of America

## Abstract

**Background:**

Health concerns for HIV-infected persons on antiretroviral therapy (ART) have moved from morbidity to the challenges of long-term ART. We investigated the effect of Zidovudine or Nevirapine on reproductive capacity across two mouse generations.

**Methods:**

A prospective mouse study with drugs administered through one spermatogenic cycle. Mouse groups (16 males and 10 females) were given Zidovudine or Nevirapine for 56 days. Males were mated to untreated virgin females to determine dominant lethal effects. Twenty females (10 treated and 10 untreated) mated with the treated males per dose and gave birth to the F_1_ generation. Parental mice were withdrawn from drugs for one spermatogenic cycle and mated to the same dams to ascertain if effects are reversible. The F_1_ generation were exposed for another 56 days and mated to produce the F_2_ generation.

**Results:**

Foetal loss was indicated in the dominant lethal assay as early as four weeks into drug administration to the males. At the first mating of the parental generation to produce the F_1_ generation, births from 10 dams/dose when the ‘father-only’ was exposed to Zidovudine (10, 100 and 250 mg/kg) was 3, 2 and 1 while it was 7, 1 and 4 respectively when ‘both-parents’ were exposed. Similarly births from the parental generation first mating when the ‘father-only’ was exposed to Nevirapine (5, 50 and 150 mg/kg) was 2, 2 and 0 while it was 6, 5 and 9 respectively when ‘both-parents’ were exposed. However, fertility was not significantly different neither by dose nor by the parental exposure. The F_1_ mice mated to produce the F_2_ generation recorded only one birth.

**Conclusion:**

The dominant lethal analysis showed foetal loss occurred when the “fathers-only” were treated while fertility was enhanced when “both-parents” were on therapy at the time of mating.

## Background

Antiretroviral drugs (ARVs) have been shown to be efficacious against the human immunodeficiency virus (HIV) for both therapy and in the prevention of mother-to-child transmission (PMTCT) of HIV [1]. With perinatal HIV transmission falling to less than 1% and life expectancy of patients on antiretroviral therapy (ART) almost similar to that of individuals uninfected by HIV, the desire of childbearing and assisted reproduction is expected to rise [2]. However, there are concerns about the effects of long-term ARVs use on the reproductive potential of people living with HIV-infection and their children. The increasing use of ARVs before, during and after pregnancy, further necessitate an investigation of the impact of these exposures. Furthermore, HIV-positive women on ARVs may become pregnant unexpectedly and expose their offspring to ARVs at very critical stages of pregnancy. Several studies have investigated the effect of ARVs on nuclear division, genome integrity and reproductive issues using cell lines, animal models and in humans, but none has investigated the effect on reproductive capacity.

Zidovudine (ZDV) have been shown to become incorporated in mammalian chromosomal DNA, preferentially incorporating into telomeric DNA and Z-DNA containing regions in Chinese Hamster Ovary (CHO) cells [3]. Sussman *et al*., [4] further showed significantly increased *HPRT* mutant frequency in human lymphoblastoid (HTK6) cell line after exposure to ZDV for 3–6 days, due mainly to significant difference in the frequency of total gene deletions [4]. Meng *et al*., [5] working with HTK6 cell line reported a significant positive correlation between ZDV-DNA incorporation and mutations in thymidine kinase (TK) that suggest ZDV incorporation had a direct role in its genotoxicity. Using Southern blot analysis, 84% of the mutants were further attributed to loss of heterozygosity, which is consistent with ZDV mode of action as a DNA chain terminator [5]. They further reported that simultaneous exposure to two NRTIs including ZDV synergistically multiplied ZDV-DNA incorporation and mutant induction frequency at both the *HPRT* and *TK* loci in HTK6 cells [6]. Escobar *et al*., [7] worked with human H9 lymphoid cells and red blood cells from perinatally exposed HIV-1-infected mothers and their infants, reporting significantly elevated glycophorin A N/N variant frequencies that persisted through one year of age in exposed children. Olivero *et al*., [8] reported that ZDV caused the accumulation of Human epitheloid Carcinoma (HeLa) cells in S-phase and altered the expression of several cell cycle genes. Divi *et al*., [9] exposed these HeLa cells to ZDV for up to 77 passages, found abnormal mitochondrial proliferation and increase in mitochondrial DNA (mtDNA) quantity early between passages 5–11. However by passages 70–77, mtDNA quantity was 65% depleted and mitochondrial membrane potential was absent [9]. These show early compensatory response was initiated before widespread mitochondrial damage and mtDNA depletion occurred. ZDV also induced both centrosomal amplification (≥2 centrosomes per cell) and aneuploidy in hamster and human cells, though there were indications the cells could still divide [10]. Centrosomal amplification has also been reported for other RTIs namely Lamivudine, Stavudine and Didanosine [11]. In spite of the centrosomal amplification, cells exposed for 24 hours were still able to complete cell division [11]. Nuclear bud formation has also been reported as a genotoxic effect of ZDV exposure for 24 hours [12]. Working with human lymphoblastoid cell lines, Olivero *et al*., [13] reported that short-term exposure to ZDV for 76 hours did not affect the nuclear division index, indicating cell viability was not altered. However, they had earlier reported that long-term exposure to ZDV through 14 passages altered the metabolic capacity of the cells through the depletion of the enzymatically active form of Thymidine Kinase 1 and altered cell cycle with cells accumulating in the S-phase [14]. In cell lines, several genotoxic changes have been recorded after exposure to ARVs.

In mice, Olivero *et al*., [15] reported a positive correlation between ZDV doses administered, the ZDV-DNA incorporation level and the proliferation of cells in the basal layer of vaginal epithelium. Mice exposed to ZDV for the last 37% of gestation period were further reported to have significant dose-dependent increases in tumor incidence and tumor multiplicity in several organs at one year of age [16]. Newborn mice were also found to have shorter chromosomal telomere after this exposure [16]. Diwan *et al*., [17] reported that exposure of mice to ZDV produced effects most prominent in the female offspring, and significant increase in lung, liver and mammary tumors. Diwan *et al*., [18] further investigated the effect of ZDV on the development of the male and female reproductive system in CD1 mice exposed between days 12 through 18 of gestation and reported no evidence of developmental reproductive toxicity using four reproductive endpoints across two generations. Using rhesus monkeys whose ZDV pharmacokinetics are similar to humans, Poirier *et al*., [19] showed ZDV-DNA incorporation into DNA of placenta and most fetal organs after short-term infusion to the mother just before delivery. Olivero *et al*., [20] demonstrated that genotoxicity measured by centrosomal amplification and micronuclei formation persisted up to three years of age in Patas monkey exposed perinatally to nucleoside reverse transcriptase inhibitors (NRTIs).

Olivero *et al*., [21], [22] demonstrated the incorporation of ZDV into DNA from ZDV-exposed individuals including adults, infants and in cord blood DNA. Olivero *et al*., [23] further demonstrated ZDV could cross the human placenta and become incorporated in DNA of placental tissue in a dose-dependent manner. Poirier *et al*., [24] reported long-term mitochondrial toxicity using samples obtained at 1 and 2 years of age in ZDV-exposed but HIV-uninfected children born to HIV-infected mothers. On human reproductive health, Frodsham *et al.,* [2] reported more HIV-discordant couples (n = 68; 93.1%) seeking assisted reproduction. Manigart *et al.,* [25] had 71 (83%) out of 85 HIV-affected couples seeking assisted reproduction being HIV-discordant couples. Coll *et al.,* [26] reported that HIV-infected women undergoing *in vitro* fertilization (IVF) treatment had a lower pregnancy rate. Vernazza [27] however reported that in 22 couples having unprotected intercourse with a short pre-exposure prophylaxis for the woman while the male partner was under completely suppressive HAART, pregnancy rates were surprisingly high (more than 50%) after three timed intercourses. Myer *et al*. [28] reviewed 11 programs across seven African countries and reported that the rate of new pregnancies was significantly higher among women receiving ART compared to women not on ART. Several factors were listed as being independently associated with increased incident pregnancy, including younger age, lower educational attainment, being married or cohabiting, having a male partner enrolled into the program, failure to use non-barrier contraception, and higher CD4 cell counts [28]. However, Manigart *et al.* [25] had reported that in couples affected by HIV, the worst results from assisted reproductive techniques were obtained when both partners were infected. Though this effect was not significant, they postulated it may be due to the small sample size (n = 14) and/or the higher mean age of the patients in that HIV-infected couples cohort [25].

Sauer *et al.,* [29] reviewed data from 10 years’ of providing fertility care to HIV-positive men with HIV-negative partners, and reported that a high rate of the infants were born premature, with preterm delivery up to 43%. Culnane *et al*., [30] had reported no difference in biometrics between controls and ARV exposed but uninfected children. As these perinatally exposed children reach their teens, they have sexual partners and express the desire to have children [31]. Irrespective of their HIV status, these ARV-treated teenagers could have sexual partners who are either HIV-positive or negative. The cumulative impact of these ARV exposures, if any, on their reproductive potential or offspring is not known. This research set out to provide some information and insight on possible events when these scenarios occur.

We investigated the impact of administering Zidovudine (ZDV) or Nevirapine (NVP) on male fertility across two generations of mice, evaluating parameters at different points along the male reproductive system. We will report the effects on sperm parameters in another paper. We here report the effect of administering Zidovudine or Nevirapine on mice reproductive potential and birth statistics. We evaluated the impact using the dominant lethal assay (DLA) and birth statistics in mice when either the “father-only” or “both-parents” had received ARVs. Mice were used to clearly establish that any effect on reproductive parameters was solely due to ART. This distinction has not been possible as most research were in HIV-infected persons often ART experienced, making it difficult to attribute effects seen to either HIV infection and/or to ART.

## Methods

### Study approval

Approval for this study was obtained from both the Institutional Review Board (IRB/09/094) and from the authorities of the Nigerian Institute of Medical Research (NIMR). The NIMR HIV treatment Centre provided Zidovudine (ZDV) and Nevirapine (NVP) paediatric syrup formulations (10 mg/ml each) used in this study. Cervical dislocation was the mouse euthanasia method applied.

### Animal husbandry and group formation

All animals were handled in accordance with the “International Guiding Principles for Biomedical Research Involving Animals” [32], which prescribes procedures for the humane treatment of research animals. Albino mice (*Mus musculus*) aged 6 weeks and above were obtained from the animal facility at the College of Medicine, University of Lagos. These were healthy albino mice that have not been used for any experimental procedures prior to this study. The albino mice were maintained in NIMR’s animal facility subject to the natural light-dark cycle during the course of the study from Sep 2010 to June 2011. Temperature and humidity of the animal house was recorded once daily using a traceable thermometer and humidity device. Mice received food and clean tap water *ad libitum*. They were housed in small groups of 5–8 mice per transparent plastic cage. Wood chippings was used as bedding and this was changed weekly. Mice were weighed and segregated into groups, allowing a maximum of 2 grams weight difference per group. These groups were then allocated to drug doses, with the heaviest mice receiving the highest drug doses. The negative control group and the three ZDV treatment groups were set-up first while the NVP treatment groups and the positive control group was set-up two weeks after to ease pressure during sampling and laboratory analysis. A total of nine hundred and sixty-two (962) albino mice were involved in this two-generational study, of which five hundred and twenty-eight (528) were born as F_1_ and F_2_ generation pups in the course of the study. Thus four hundred and thirty-four (434) mice was the input sample size for the study.

### Study design and treatment procedures

**Figure 1:**
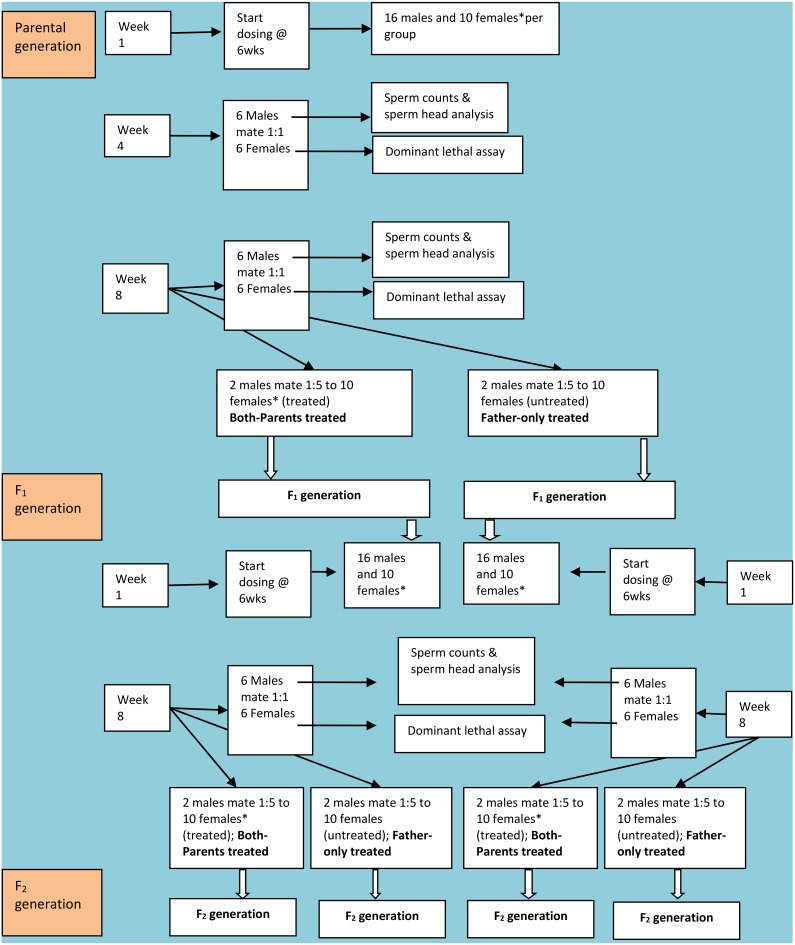
Diagrammatic representation of the study design.

Mice were dosed once daily by gavage for 8 weeks to cover one complete spermatogenic cycle which is approximately 56 days in mice [33]. A diagrammatic representation of the study design is shown as Figure 1. Three doses per drug were administered by gavage to simulate the oral administration of ARVs in humans. For Zidovudine 10, 100 and 250 mg/kg were administered while for Nevirapine 5, 50 and 150 mg/kg were administered. Drug doses were selected considering the doses used in mice studies reported in the literature and the established human dosage. Individual mouse weight was determined weekly during the first 2 weeks of the study and thereafter weights were ascertained at least fortnightly for the non-pregnant mice. For pregnant dams, individual weights were determined weekly. The average weight of each weight-matched group was then used to determine the quantity of drug administered to the entire group per week or fortnightly.

There were six treatment groups and two (negative and positive) control groups. Ethylmethylsulfonate (EMS) was administered as the positive control drug. Each dose was administered daily to 16 male and 10 virgin female mice for eight weeks (56 days). The sample size was selected considering the requirements outlined in OECD guide 416 [33] and the 3Rs policy (Replacement, Refinement and Reduction policy) [34]. At the end of week three, six males per dose were randomly selected and mated (1∶1 cohabitation for seven days) to six untreated virgin females. The males continued to receive their ARV drug doses through week four. At the end of seven days co-habitation and four weeks of drug administration, the mated untreated females were separated and used for the dominant lethal assay (DLA) [35], [36]. The males were euthanized to obtain sperm from the cauda epididymis for analysis [36]. At the end of seven weeks of drug administration, six males were randomly selected again and mated (1∶1 cohabitation for seven days) to six untreated virgin females. These males continued to receive their doses of the ARV drug until week eight. At the end of seven days co-habitation and eight weeks of drug administration, the mated untreated females were separated and used for the DLA while the males were used for sperm analysis. Four treated males per dose were now remaining at the end of week eight. Two treated males were each mated (1∶5 cohabitation for 10 days) to 10 untreated females while the other two mated (1∶5 cohabitation for 10 days) to the 10 treated females co-administered ARVs for eight weeks. They were mated by co-habitation for 10 days without checking for vaginal plugs. The treated females had received the same drug and dosing that their male partners’ had received. These treated and untreated females per dose produced the F_1_ progeny having “both-parents” or the “father-only” treated parental groups respectively, simulating human situations when both-partners or the male partner only is on ART. The dams from the “both-parents” treated groups continued to receive their drugs during and after pregnancy until the offspring were weaned 4 weeks after birth. The mating and sampling at week four represent mid-term effects while the mating and sampling at week eight represent effects at full-term. The negative control group did not receive any drug while the positive control group received one-time dose of 480 mg/kg ethylmethanesulfonate by gavage. Both the negative and positive control groups were subjected to the same mating procedures at week three, seven and eight.

The four males per treatment dose that sired the F_1_ mice were kept for another 8 weeks without administering the ARVs. Thereafter, they were mated again (repeat mating) to the same set of treated and untreated females to ascertain if the effects of the drug administration could resolve after the drug was withdrawn for one full spermatogenic cycle. Thus at repeat mating, the males had been withdrawn from ARVs and only the dams from the “both-parents” treated groups were still on ART having just weaned their pups.

The F_1_ study continued with only the progeny from the 10 mg/kg ZDV and 5 mg/kg NVP treatment groups, and separately for mice descendent from the “both-parents” or “father-only” treated arms per drug. Surviving F_1_ progeny were weaned at age four weeks. The sexes were separated and two weeks period given for all dams to wean. Most of the four F_1_ groups had surviving progeny less than the 16 males and 10 females sample size used per group. The available number of progeny was used. At age≥6 weeks, the eight weeks-dosing procedure commenced for the F_1_ mice. From the F_1_ progeny of the “both-parents” treated groups, surviving males and females began the eight weeks-dosing protocol previously used to dose the parental generation. However, DLA was done for only the ZDV groups at mid-week and then discontinued. Mating (1∶5 co-habitation for 10 days) to produce the F_2_ generation was done after eight weeks of drug administration. Similarly F_1_ progeny of the “father-only” treated groups began the eight weeks-dosing protocol. DLA was also done for only the ZDV groups at mid-week and then discontinued. Mating (1∶5 co-habitation for 10 days) to produce their F_2_ generation was done after eight weeks of drug administration.

### Sampling and laboratory analysis

The dominant lethal assay (DLA) was performed using the untreated virgin females mated to the treated males. After their one week 1∶1 co-habitation with the treated males, the females were separated. The females were sacrificed in the early second half of gestation (13 days from the mid-week of mating). After the dams were euthanized, the uteri were removed and weighed. The uterine contents were then exposed. The number and uterine position of any re-absorptions and/or foetus (live or dead) were recorded. The total number of implants was also recorded.

Close to delivery, a birth register was opened and the dams were checked at least once every 6 hours to record births and the birth weights. The pups were not handled while weighing but rather lifted using their beddings. A top-loading weighing balance (Ohaus Scout Pro) was used. Birth statistics were collated and compared. These include fertility (number of pregnancies), gestation periods, litter sizes, birth weights and pup survival proportions (as number of surviving pups at weaning 4 weeks after birth). In mice birth weights are often a function of the litter size, thus residuals were obtained from the expected birth weights per litter size reported by Enzmann and Crozier [37] and compared. The residuals were calculated as the average group birth weight minus the expected as a percentage of the expected.

### Data management and analysis

The Fisher exact chi-square test, the Wilcoxon rank sum test, Analysis of Variance (ANOVA) and the Kruskal-Wallis test were the statistical tests applied as appropriate, depending on the data distribution and variance between groups. A p-value less than 0.05 was regarded as significant.

## Results

The median weight (± standard deviation; range) of the parental generation mice at initiation of treatment was 26.0 gm (±3.5; 19–38) for the males and 24.0 gm (±1.3; 22–27) for the females. And the median weight of the F_1_ mice at initiation of treatment was 16.5 gm (±4.1; 10–24) for the males and 15.3 gm (±2.6; 11–20) for the females. The median temperature (± standard deviation; range) in the animal house for the 10 months study duration was 30.8°C (±2.2; 25.6–36.2) while the median humidity was 65% (±14; 20–90).

### Dominant lethal assay (DLA) analysis

The DLA analysis after mating (1∶1) at week four showed that the negative control (NC) group had one pregnant dam. ZDV 100 mg/kg and NVP 5 mg/kg groups had three pregnant dams each while NVP 150 mg/kg had one pregnant dam. In addition, the NVP 150 mg/kg and 50 mg/kg groups each had one dam with heavy arterial lining of the uterus and/or with uterine fluids (Table 1). At mid-term, only 4 out of the 8 groups had implants but there was no pattern between the number of pregnant dams or number of implants per pregnancy and the drug concentrations (Table 1). Foetal loss was indicated in 2 of the 3 NVP groups.

**Table 1:** Dominant lethal assay (DLA) after males were treated with Zidovudine or Nevirapine for four weeks.

Drug	Dose (mg/kg)	Sample size (n)	aPregnant (n)	DLA Implants (n)				
				cSize	Dead		Resorbed	
None	None	6	1	8.0		0		0
EMS	480	6	b0/1	-		-		-
ZDV	10	6	-	-		-		-
	100	6	3	8.3		0		0
	250	6	-	-		-		-
NVP	5	6	3	6.0		0		0
	50	6	b0/1	-		-		-
	150	6	b1/2	7.0		0		0

DLA simulates when the “father-only” is ARV treated.

EMS = Ethylmethylsulfonate (one-time dose); ZDV = Zidovudine; NVP = Nevirapine.

a Number of pregnancies observed after 1∶1 mating.

b Number of pregnancies before/after adding dams with heavy arterial linings to the pregnancy count. Dams with heavy arterial lining of the uterus and/or uterine fluids indicate fertile mating had occurred but was aborted early.

c Number of implants per pregnant dam or the average from all pregnant dams in the group.

Statistical analysis could not be performed because of several missing values.

After mating (1∶1) at week 8, DLA analysis for the NC group showed that there were four pregnant dams with average implant size of 6.8 (Table 2). At full-term, all the ZDV groups had implants while none of the NVP groups had implants. The ZDV groups have two to three pregnant dams per dose. The presence of heavy arterial lining of the uterus and/or with uterine fluids was observed in all treatment groups except for the ZDV 250 mg/kg group. Only the ZDV 100 mg/kg group had one pregnant dam presenting with both dead and resorbed implants while the ZDV 250 mg/kg group had resorbed implants in one pregnant dam. Two out of the three ZDV groups (100 and 250 kg/mg groups) had higher number of implants per pregnancy compared to the untreated control (Table 2). Though none of the NVP groups had a pregnant dam at week 8, the heavy arterial lining of the uterus and/or with uterine fluids was observed in two to three dams per group indicating that fertile mating may have occurred but all were aborted. Among the F_1_ groups, DLA was done for the ZDV groups’ mid-term and no pregnancy was recorded. Hence the DLA analysis for the F_1_ groups was discontinued.

**Table 2:** Dominant lethal assay (DLA) after males were treated with Zidovudine or Nevirapine for eight weeks.

Drug	Dose (mg/kg)	Sample size (n)	aPregnant (n)	DLA Implants (n)					
				cSize	Dead			Resorbed	
None	None	6	4	6.8		0	1.0		
EMS	480	6	2	8.0		0	8.0		
ZDV	^d^10	6	b2/4	6.0		0	0		
	^d^100	6	b3/4	8.3		1.0	1.0		
	250	6	3	9.0		0	4.0		
NVP	5	6	b0/2	-		-	-		
	50	6	b0/2	-		-	-		
	150	6	b0/3	-		-	-		

DLA simulates when the “father-only” is ARV treated.

EMS = Ethylmethylsulfonate (one-time dose); ZDV = Zidovudine; NVP = Nevirapine.

a Number of pregnancies observed after 1∶1 mating.

b Number of pregnancies before/after adding dams with heavy arterial linings to the pregnancy count. Dams with heavy arterial lining of the uterus and/or uterine fluids indicate fertile mating had occurred but was aborted early.

c Number of implants per pregnant dam or the average from all pregnant dams in the group.

Differences in number of aborted pregnancies at week 4 versus week 8 was not significant.

### Mice fertility

The NC group had an average of four out of ten dams giving birth. After eight weeks of treatment, the first mating to produce the F_1_ generation resulted in a total of 10 and 32 pregnancies for all the “father-only” and the “both-parents” treated groups respectively (Table 3). Only the NVP 150 mg/kg “father-only” treated group recorded no pregnancy at the first mating. The ZDV 10 mg/kg group recorded seven pregnant dams out of ten and had the most pregnancies among ZDV “both-parents” treated groups. The NVP 150 mg/kg recorded nine pregnant dams out of ten among to top the NVP “both-parents” treated groups (Table 3).

**Table 3:** Number of Pregnancies in relation to dosing and parental treatment at the first and repeat mating to produce the F_1_ generation.

Drug	Dose (mg/kg)	Dam Sample size (n)	aPregnancies at First mating		bPregnancies at Repeat mating	
			Father-only	Both-parents	Father-only	cBoth-parents
ZDV	10	10	3	7	2	2
	100	10	2	1	2	1
	250	10	1	4	1	2
NVP	5	10	2	6	2	0
	50	10	2	5	5	4
	150	10	0	9	2	2
**Total**			**10**	**32**	**14**	**11**

Four males per dose mated with five females each to give a total of twenty females per dose. Ten of these females were untreated (“father-only” treated groups) while the other ten had received the same dose as the males (“both-parents” treated groups) for 56 days before mating. Harem mating was done by co-housing one male and five females for 10 days without checking for vaginal plugs.

ZDV = Zidovudine; NVP = Nevirapine.

a Pregnancies observed when all males were on therapy at the time of mating.

b Pregnancies observed when all males have been withdrawn from therapy for one spermatogenic cycle (56 days) before mating the same set of dams again.

c Both parents had been on therapy but at repeat mating, only the dams were currently on therapy as the males had been withdrawn for one full spermatogenic cycle.

Using the Fisher’s Exact Chi-squared test, there was no significant different in the pregnancies recorded by dose and also no significant difference in pregnancies observed for the “father-only” and “both-parents” groups.

During the repeat mating to produce the second F_1_ generation, after the males had been withdrawn from treatment for another 8 weeks, a total of 14 and 11 pregnancies were recorded for the “father-only” and the “both-parents” treated groups respectively (Table 3). The treatment groups had more pregnancies at the first mating (n = 42) than at the repeat mating (n = 25). However at both times, the distribution of pregnancies was not significantly different either by dose or by parental treatment. At the repeat mating, two pregnant dams in labour died before giving birth and were not counted. One dam died from the NVP 5 mg/kg “both-parents” treated group and another from the 50 mg/kg “father-only” treated group.

After eight weeks of treatment, the F_1_ mice were mated and recorded only one pregnancy. This pregnancy was from the NVP 5 mg/kg “both-parents” treated F_1_ group. This F_1_ group was descendent from the NVP 5 mg/kg “both-parents” treated parental group (Table 4).

**Table 4:** Number of Pregnancies obtained from the F_1_ treatment groups.

	Number of Pregnancies (Number of dams mated)			
P generation exposure →	Progeny from “Father-only” treated group		Progeny from “Both-parents” treated group	
F_1_ Group exposure →	Father-only	Both-parents	Father-only	Both-parents
ZDV 10 mg/kg	0 (10)	0 (2)	0 (10)	0 (10)
NVP 5 mg/kg	0 (10)	-	0 (10)	1 (8)

### Gestational period

The NC group had an average gestation period of 26 days. Shorter gestation periods were observed at both the first and repeat mating for all parental treatment groups (Table 5) ranging from 17–25 days. The shortest gestation of 17 days was recorded for ZDV 100 mg/kg “both-parents” treated group while the longest gestation of 25 days was recorded for ZDV 250 mg/kg “both-parents” treated group. A gestation period of 24 days was determined for the only pregnancy recorded among the mated F_1_ groups.

**Table 5:** Gestation period in relation to dose and the parental mouse treatment.

Drug	Dose (mg/kg)	Dam Sample size (n)	aGestation period afterFirst mating (Days ± SD)		bGestation period afterRepeat mating (Days ± SD)	
			Father-only	Both-parents	Father-only	cBoth-parents
None	None	10	26.0±1.4	26.0±1.4	26.0±1.4	26.0±1.4
EMS	480	10	22.4±2.3	24.4±4.2	20.0†	24.0±4.4
ZDV	10	10	20.7±2.9	23.1±2.8	17.5±0.7	20.5±4.9
	100	10	22.0±4.2	17.0†	20.5±0.7	18.0†
	250	10	22.0†	25.0±3.6	20.0†	20.5±2.1
NVP	5	10	21.5±3.5	18.8±1.0	24.5±2.1	-
	50	10	22.0±0.0	19.8±4.4	21.4±4.5	20.5±3.0
	150	10	-	18.9±1.9	22.5±2.1	20.5±3.5

Four males per dose mated five females each to give a total of twenty dams per dose. Ten of these dams were untreated (“father-only” treated groups) while the other ten had received the same dose as the males (“both-parents” treated groups) for 56 days before mating. Harem mating was done by co-housing one male and five females for 10 days without checking for vaginal plugs. ZDV = Zidovudine; NVP = Nevirapine; EMS = Ethylmethylsulfonate (one-time dose); SD = Standard Deviation.

a Average gestation period obtained for dams mated to males on therapy at the time of mating.

b Average gestation period obtained for dams mated to males previously treated but have not received ARVs for another spermatogenic cycle (56 days) to check if effects could resolve. Thus only the females were still on therapy at the repeat mating.

c Both parents had been on therapy but at repeat mating, only the dams were currently on therapy as the males had been withdrawn for one full spermatogenic cycle.

† Single birth recorded, thus SD could not be calculated.

Using ANOVA, significantly shorter gestation periods was observed only for the NVP “both-parents” treated groups at first mating and for the ZVP “father-only” treated groups at repeat mating when the treatment groups were compared to the untreated control.

### Litter size and Birth weights

The average number of pups per litter (litter size) for the untreated controls was 6 pups and their average litter birth weight was 1.41 g. Among the treated groups, litter size ranged from 2–10 pups per dam while the average litter birth weight ranged from 1.15–1.63 g. Litter size varied across the treated groups but the differences between the NC group and all treated groups at both the first and repeat mating were not significant. Most treatment groups had positive residuals (0.8–25.9%) from their expected birth weight, however the ZDV 250 mg/kg “father-only” treated group recorded negative residuals (–2.4%) from the expected. The single pregnancy recorded among the mated F_1_ groups had three pups with average birth weight of 1.57 g and a positive residual (27.6%) from the expected birth weight.

### Proportion of Surviving Pups and sex ratios

Of the 24 pups born to the NC group, 17 (70.8%) survived till weaning at age 4 weeks. A total of 61 and 205 pups were born to the “father-only” and the “both-parents” treated groups respectively. The survival rates determined at weaning for pups born to the treated groups ranged from zero to 100% (Table 6). Pup deaths in ZDV 250 mg/kg group were due to cannibalism within 1–2 days of birth. Deaths occurring in ZDV 10 mg/kg and NVP 5 mg/kg groups had carcass without bodily signs of cannibalism. The difference in both the average and median survival proportion (%) of the pups born to the “both-parents” treated groups (50.4 and 47.7 respectively) were not significant compared to those of the “father-only” treated groups (41.0 and 31.8 respectively). Sex ratios were determined and did not differ from the expected male to female (1∶1) sex ratio.

**Table 6:** Proportion of surviving pups and their sex ratios in relation to dose and parental treatment.

Drug	Dose (mg/kg)	aFather-only treated			aBoth-parents treated		
		Pups born (n)	Pups Survived (n)	Female to Male ratio	Pups born (n)	Pups Survived (n)	Female to Male ratio
None	None	24	17	8∶9			
ZDV	10	22	7	2∶5	50	27	14∶13
	100	10	8	5∶3	7	7	5∶2
	250	4	0	-	28	0	-
NVP	5	10	2	0∶2	39	14	8∶6
	50	15	11	9∶2	29	12	7∶5
	150	0	-	-	52	37	22∶15
bTotal (n)		61	28		205	97	

Proportion of surviving pups was determined while weaning the pups at age 4 weeks.

ZDV = Zidovudine; NVP = Nevirapine.

a Four males from each dose mated five females each to give a total of twenty dams per dose. Ten of these dams were untreated (“father-only” treated groups) while the other ten had received the same dose as the males (“both-parents” treated groups) for 56 days before mating. Harem mating was done by co-housing one male and five females for 10 days without checking for vaginal plugs.

b We compared the survival proportion of the treatment groups to that of the untreated control group (70.8%) using the binomial test. The proportions of surviving pups were significantly different for most treatment groups except for that of the ZDV 100 mg/kg at both first and repeat mating; for NVP 50 mg/kg at first mating and for NVP 150 mg/kg at repeat mating.

The 3 pups from the single birth among the F_1_ groups were euthanized before weaning at four weeks as the project was winding down. Thus data on survival proportion and sex ratio was not obtained.

## Discussion

### Dominant Lethal assay (DLA) Analysis and foetal loss

In some mated but non-pregnant dams, heavy arterial lining and/or bloody uterine fluids were observed. This indicates that fertile mating that could have resulted in pregnancies may have occurred but were aborted. These observations in dams from all parental Nevirapine groups and two Zidovudine groups at week 8 indicated fertile mating that could have resulted in pregnancies had occurred. However, while some foetuses among the Zidovudine groups were aborted, the dams that received Nevirapine had aborted all foetuses. Foetal loss was thus observed in untreated dams mated to males exposed to Nevirapine or Zidovudine for one full spermatogenic cycle. In mice foetal loss is thus associated with administering Zidovudine or Nevirapine to the father-only, and was particularly prevalent among the Nevirapine treated groups.

### Fertility

At first mating to produce the F_1_ generation, fertility was enhanced among most “both-parents” treated groups while fertility was comparatively lower when the “father-only” was on therapy. The “both-parents” treated groups had more pregnancies (3.2 times), consequently more pups were born (3.3 times) and also more pups surviving at weaning (3.5 times). However, none of these endpoints were significantly different, more so because it cuts across two different drugs administered in various doses. Comparatively, fertility was reduced when the “father-only” was on therapy. They had lower number of pregnancies and pups, in addition to the few pregnancies recorded in the DLA analyses, The DLA analyses performed was essentially a “father-only” treated scenario. Comparing the number of pregnancies among the “both-parents” treated groups at the first and repeat mating suggests that the apparent boost in fertility only obtains when both parents were currently on ARV therapy at the time of mating. Similar comparison among the “father-only” treated groups at first and repeat mating show that when the ARVs were withdrawn for another spermatogenic cycle, there was no significant improvement in fertility. This suggests that the impact of the ARVs may not be reversible, at least immediately.

Few animal studies on the effects of ARV therapy on reproductive capacity have been carried out and we found none to compare our results with. Diwan *et al*., [18] was the closest, investigating the developmental reproductive toxicity of the ARVs by exposing perinatally during the gestational period of sexual differentiation and found no difference in endpoints such as anogenital distance, onset of testicular descent etc.

Thus we looked for correlate studies among humans to compare with. Clearly we cannot directly extrapolate these results in mice to human studies. Nevertheless, there are some noteworthy correlates from human studies. First, it is mainly HIV sero-discordant couples that present at assisted reproduction or fertility clinics seeking assisted fertility procedures [2], [25] and we have shown fertility appeared reduced when only the male mouse was on therapy. Secondly, some fertility treatments are ethical only with pre-exposure ARV prophylaxis for the uninfected spouse such as prior to timed unprotected intercourse. This creates a scenario where both spouses are on therapy and had surprisingly high success rate (>50%) recorded in a human study after three timed intercourses [27]. In addition, a review of 11 programs across seven African countries reported significantly higher rates of new pregnancies among women receiving ART compared to women not on ART, which was independently associated with having a male partner also enrolled in the programme [28]. No behavioural or biomedical mechanisms was reported in literature for this phenomenon of increased fertility [28]. Clearly more studies are required to elucidate on this. We can only conclude that in mouse, treatment with Zidovudine or Nevirapine apparently increased number of pregnancies when “both-parents” were ARV-treated but the offspring from these pregnancies had greatly reduced number of pregnancies in the second (F_2_) generation.

### Gestational period and pup survival

In this study comparison to the untreated controls showed shorter gestation periods were recorded for all treatment groups, though these were not significant. Since there are no mice studies to make comparison to, we note that this finding agrees with the report of a high rate of preterm delivery (43%) among HIV-affected couples [29]. Shorter gestation periods in the mouse treatment groups of this study are clearly attributed to the administration of Zidovudine or Nevirapine.

Pup survival proportions determined at weaning were varied. Similarly the death scenarios of pups were varied. Some pups were cannibalised by their mothers within hours/days of birth, while others died with no signs of cannibalism on their carcass often weeks from birth. Strain and hormonal differences have been shown to influence pup-killing behaviour by female mice [38], [39]. Furthermore, females who give birth to deformed offspring were found more likely to consume their deformed offspring or dead young than their living healthy siblings [40], [41]. Schardein *et al*., [41] reported that 21% of live congenitally malformed offspring were killed and consumed in contrast to only 3% of normal live offspring. Similarly among stillborn offspring, the females consumed 63% of the congenitally malformed ones but only 19% of the normal ones [41]. The same stock of animals were used for this study, thus hormonal variation and/or congenital defects could be responsible for this infanticide behaviour. With the ARVs tested in this study being DNA-reactive, congenital defects are a highly probable cause particularly as none of 32 pups born to groups administered the highest dose of Zidovudine survived. Pups that died among the least doses administered for each drug had no bodily signs of cannibalism on the dead carcass and could be due to congenital defects. In this study, any congenital defects would be metabolic or genetic as no physical deformity was observed in any pups at birth or during weaned.

### Litter size/implants, birth weights and reversibility of effects

Lack of significant difference between the untreated control and the treatment groups in the litter sizes and in the birth weights of pups indicates that Zidovudine or Nevirapine may not have an impact on litter size and birth weights in mice. In humans, an early report [30] had similarly found no difference in biometrics between controls and ARV-exposed but HIV-uninfected children.

### Further research and study limitations

A key further research question from this study is the elucidation of the mechanism(s) enhancing fertility when both parents were treated with antiretroviral drugs. The reasons for the shorter gestation period and foetal loss here attributed to ARV therapy remains to be elucidated.

Lack of strain information on the mouse used is a limitation of this study. Our focus on the male reproductive system in this preliminary study limited the explanation of our findings as no parameter for the female reproductive system was assessed.

## Conclusion

We conclude that in mice, treatment with Zidovudine or Nevirapine is associated with foetal loss and an apparent increase in fertility when the males-only or both-parents are treated respectively.
